# Population-based prevalence survey of tuberculosis in the Tigray region of Ethiopia

**DOI:** 10.1186/1471-2334-13-448

**Published:** 2013-09-28

**Authors:** Gebretsadik Berhe, Fikre Enqueselassie, Elena Hailu, Wondale Mekonnen, Tsigemariam Teklu, Ataklti Gebretsadik, Rezene Berhe, Tewodros Haile, Abraham Aseffa

**Affiliations:** 1 Armauer Hansen Research Institute, Addis Ababa, Ethiopia; 2 School of Public Health, Addis Ababa University, Addis Ababa, Ethiopia; 3 College of Veterinary Medicine, Mekelle University, Mekelle, Ethiopia; 4 Tigray Health Bureau, Tigray, Ethiopia; 5 Axum University, Axum, Ethiopia

**Keywords:** Bacteriologically confirmed, Cross-sectional, Pulmonary tuberculosis, Tigray region, Ethiopia

## Abstract

**Background:**

Population based prevalence survey is an important epidemiological index to measure the burden of tuberculosis (TB) disease and monitor progress towards TB control in high burden countries like Ethiopia. This study was aimed to estimate the prevalence of bacteriologically confirmed pulmonary tuberculosis (PTB) in the Tigray region of Ethiopia.

**Methods:**

Sixteen rural and urban villages were randomly selected in a stratified multistage cluster sampling. Individuals aged 15 years and older were screened by symptom inquiry for PTB. Those individuals who were symptomatic of PTB provided two sputum samples for smear microscopy, culture and molecular typing.

**Results:**

The study covering 4,765 households screened a total of 12,175 individuals aged 15 years and above. The overall weighted prevalence of bacteriologically confirmed PTB in the Tigray region of Ethiopia was found to be 216/100,000 (95% CI: 202.08, 230.76) while the weighted prevalence of smear-positive PTB was 169/100,000 (95% CI: 155.53, 181.60). The prevalence of bacteriologically confirmed TB was higher amongst males (352/100 000; 95% CI: 339.05, 364.52) than females (162/100 000; 95% CI: 153.60, 171.17) and among rural (222/100,000; 95% CI: 212.77-231.53) as compared to urban residents (193/100,000; 95% CI: 183.39-203.59).

**Conclusions:**

This study found a relatively higher prevalence smear-positive PTB in the region than in a same period nationwide survey and identified a significant number of undetected PTB cases. The urgency for improved TB case detection and intensified community awareness is emphasized.

## Background

Tuberculosis (TB) is a major global health problem. All countries are affected, but most cases occur in the 22 so-called high-burden countries (HBCs) that account for about 80% of the world’s TB cases [1]. TB control activities require regular direct measurement of the absolute burden of disease to monitor trends and improve understanding of the epidemiology of TB in the target area [2]. Epidemiological information on TB is also vital for the planning of control strategies and service delivery systems [3-5] especially in countries where there is considerable uncertainty about the number of TB cases and deaths, due to incomplete coverage or absence of surveillance systems [5,6]. However, in many HBCs, notification systems do not record all cases and vital registration systems are either absent or of such poor quality and coverage that TB statistics is often unreliable [4,6].

In many resource poor settings, estimates of the prevalence of pulmonary tuberculosis (PTB) are based on symptom screening at health facilities followed by smear microscopy with or without culture examination of eligible individuals [3,5,7-14]. Although TB is one of the leading causes of morbidity and adult deaths in Ethiopia, there are very few reports [5,7-10,15] on the magnitude of TB from population-based surveys. Most of the TB data come from health institution-based case notifications that often lack completeness and consistency [5]. In a large country like Ethiopia where there is socioeconomic, lifestyle and environmental variations among the different regions, a national TB prevalence survey cannot be used to estimate TB prevalence values for the different regions in the country. The present study was undertaken to estimate the prevalence of bacteriologically confirmed PTB amongst the adult population of the Tigray region through a population-based survey.

## Methods

### Study setting

Tigray Region is the northernmost of the nine regions of Ethiopia and it is the homeland of the Tigray people. Excluding Mekelle town, the state capital, there are seven administrative zones: comprising a total of 47 districts and 673 *Kebelles* (smallest administrative unit) [16]. Based on the 2007 census projection, Tigray has an estimated total population of 4.8 million people over an area of 50,078.64 square kilometers). Most (80.5%) of the population live in rural, while 19.5% are urban dwellers [16]. According to the 2008 Health and Health Related Indicators published by the Federal Ministry of Health [17]. Tigray region had 15 hospitals, 123 health centers, 182 health stations and 614 health posts. This study was carried out in 16 randomly selected districts including six urban and ten rural districts.

### Study design and population

A population-based cross-sectional study design was employed to estimate the prevalence of bacteriologically confirmed PTB. The study was conducted in the period between March to August 2011.

Source populations were all adult individuals whose age were fifteen years and above and permanently living in the Tigray region of Ethiopia. Sample populations were individuals in the source population identified by the sampling procedure and the study subjects were sample populations who met criteria of a TB suspect. The criteria for inclusion in the study were providing written informed consent, living in the selected house permanently for at least 2 months prior to the study and age of at least 15 years or above at the time of the survey.

### Sample size and sampling techniques

The sample size of the prevalence survey was calculated based on the following assumptions using epiInfo version 3.2.2, 2004. For a conservative TB prevalence estimate of 0.25% extrapolated from the WHO report [18], a margin of error of 0.1% at 95% confidence interval (CI), a design effect of 2, and non-response rate of 10%, the required sample size was calculated as 9,359 persons. However, to improve precision, 12,175 adults were sampled.

**Figure 1:**
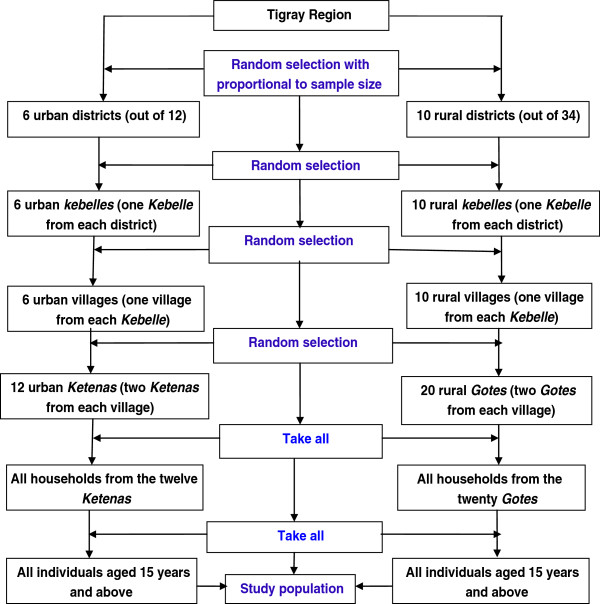
Sampling protocol of the prevalence survey in Tigray region, 2011.

A stratified multistage cluster sampling procedure was adopted to recruit the study subjects (Figure 1). For urban population, at the first stage, five towns (out of 12) (A town in the Region is defined as having a population of greater than 10,000 population) were randomly selected. One urban district, Korem, was added to the five town districts by simple random sampling making them altogether six districts. At the second stage, one *Kebelle* from each town and one village within each selected *Kebelle* was selected randomly. Then two *Ketenas/Gotes* from each village were randomly selected and then all adult individuals living in these selected *Ketenas* were sampled. In the rural areas, ten districts were randomly selected. At the second stage, one *Kebelle* within each district and one village within each selected *Kebelle* were randomly selected, respectively. Finally two *Gotes* were randomly selected from each village and all adult persons in the selected *Gotes* were sampled for the study. A cluster in this study was defined a small area called *Gote* or *Ketena* (sampling unit for the survey) which consisted of 150 to 200 households.

### Data collection procedure

A total of 16 nurses and 16 laboratory technicians working in the respective districts were recruited and trained in basic interview techniques and with the objective of the study. The data collectors were assigned to the study *Kebelle* in sixteen groups, with one nurse and one laboratory technician in each group. They then made house-to-house visits. The nurses interviewed the head of each of the households using a pre-tested and structured symptom screening questionnaire if anyone in the household aged ≥ 15 years has chronic cough (cough occurring for more than two weeks of duration) with or without chest symptoms, namely: chest pain for ≥ 1 month; fever for ≥ 1 month; and haemoptysis at any time. They also documented if any member of the household was under treatment for TB.

When the team encountered symptomatic individuals, the laboratory technicians collected two sputum samples ('spot-morning’) from each symptomatic TB suspect, including those individuals who were already on treatment, in sterilized bottles — the first specimen during the interview and the second early the next morning. Information concerning the socio-demographic and clinical characteristics was also collected from symptomatic individuals. The sputum samples were transported to the district health center in an ice box to be examined for AFB and stored temporarily at 4°C. The sputum samples were then transported to the Tigray regional laboratory within two days of collection and at the regional laboratory samples were kept for a maximum of two months duration in -20°C until they were transferred to the Armauer Hansen Research Institute (AHRI) at Addis Ababa on ice box for culture and characterization of isolates.

### Laboratory tests

All sputum samples were tested for acid fast bacilli (AFB) smear-microscopy using Ziehl Neelsen (ZN) stain. At AHRI, all morning and spot sputum samples from the same individual were pooled. All specimens were cultured and positive isolates were characterized by deletion typing for speciation of mycobacteria according to standard procedures [19,20].

A prevalent TB case was defined in this survey as an individual whose sputum was positive for acid fast bacilli by ZN microscopy and/or growth of *M. tuberculosis* (MTB) by culture examination [21].

Data were checked and double entered and stored in Microsoft Access 2007 software program. Univariate analysis was conducted using STATA/SE 11.0 for windows (STATA Corporation, College Station, TX, United States of America) and confidence intervals (CI) were calculated from the estimated population proportion.

The study was approved by the Institutional Review Boards of the Addis Ababa University and AHRI. Written informed consent was obtained from study participants. Written informed assent was secured from study subjects who were between 15–18 years of age in addition to written consent from their parents or guardians.

## Results

### Socio-demography of the study population

The prevalence survey was carried out in sixteen districts. The source population consisted of 20,157 individuals of whom 53.7% (10,861) were female and 46.3% (9,352) male. A total of 4,765 households was screened evaluating 12,175 individuals aged 15 years and above for PTB through symptom interviews. The average number of study participants per cluster was 760.9 (range 978–1587). Of the total adult study participants, 5401 (44.4%) were male and 6774 (55.6%) female with male to female sex ratio of 0.8:1. The size of the screened population per district ranged from 1,587 in Ahferom to 978 in the Korem district (Table 1).

**Table 1:** Distribution of study population by age, sex, and district in the Tigray region, 2011

**District**	**Study population**	**Females**		**Males**		**Adults**
		**< 15 yrs**	**≥ 15 yrs**	**< 15 yrs**	**≥ 15 yrs**	**(≥ 15 yrs)**
Adigrat	1194	198	456	225	315	771
Adwa	1287	247	493	240	311	804
Ahferom	1587	390	422	382	374	796
Alamata	1125	165	423	217	316	739
Asgede Tsimbla	1485	330	419	354	375	794
Atsbi Wenberta	982	209	355	196	250	605
Axum	1248	226	451	233	338	789
Enderta	1123	203	406	191	310	716
Kafta Humera	1421	261	459	240	467	926
Korem	978	173	396	167	253	649
Laelay Maichew	1103	239	379	207	284	663
Offla	1065	302	317	225	336	765
Raya Azebo	1275	268	429	242	336	765
Saesie Tsaedaemba	1477	331	474	293	397	871
Setit Humera	1266	218	440	187	432	872
Tahtay Koraro	1541	327	455	352	420	875
**Total**	**20157**	**4087**	**6774**	**3951**	**5401**	**12175**

### Prevalence of pulmonary TB

Among the total 12,175 screened individuals in the studied districts, 350 PTB suspects were identified. From the 350 study subjects, the survey found 30 culture and/or smear-positive individuals out of which 24 were smear-positive and 27 were culture positive (Table 2). Three smear-positive cases did not yield isolates while six AFB negative cases were positive on culture. On the other hand, the survey identified 30 existing cases of PTB in the community, among which 21 reported that they were still taking anti-TB treatment during the survey. Among the 21 previously known PTB cases who were taking anti-TB treatment during the survey, 11 cases were still smear and/or culture positive, 3 were smear-positive but culture-negative while the other 7 were smear and culture negative. All isolates were confirmed to be MTB by RD9 deletion typing.

**Table 2:** Distribution of PTB by districts in the Tigray region, 2011

**District**	**Subject screened**	**No. AFB positives**	**No. MTB positives**	**Rate per 100, 000**	**95% CI**
Adigrat	771	3	4	518.81	(483.54, 554.08)
Adwa	804	3	3	373.18	(339.70, 406.57)
Ahferom	796	0	0	0.00	0.00
Alamata	739	2	3	405.95	(370.55, 441.36)
Asgede Tsimbla	794	0	0	0.00	0.00
Atsbi Wenberta	605	1	1	165.29	135.69, 194.89)
Axum	789	2	3	380.23	(346.35, 414.10)
Enderta	716	0	0	0.00	0.00
Kafta Humera	926	1	1	107.99	(88.00, 127.98)
Korem	649	1	1	154.08	(126.31, 181.86)
Laelay Maichew	663	1	1	150.83	(123.59, 178.07)
Offla	540	0	0	0.00	0.00
Raya Azebo	765	3	3	392.16	(357.56, 426.75)
Saesie Tsaedaemba	871	1	4	459.24	(426.15, 492.34)
Setit Humera	872	3	3	334.04	(312.51, 375.57)
Tahtay Koraro	875	3	3	342.86	(93.20, 135.37)
**Total**	**12,175**	**24**	**30**	**246.41**	**(238.75, 254.06)**

Symptom screening found that out of the 350 PTB suspects identified through a house-to-house visit, 97.14% (340/350) suspects had a chronic cough with one or more of the other symptoms (weight loss; excessive night sweat; chest pain for ≥ 1 month; fever for ≥ 1 month; and haemoptysis) while the remaining 2.86% (10/350) individuals had only the other symptoms without a chronic cough. All of the PTB positive cases were diagnosed from those suspects who had a chronic cough with one or more of the other symptoms.

Therefore, the overall weighted prevalence (culture and/or smear) of PTB in Tigray region of Ethiopia was found to be 216 per 100,000 (95% CI: 209.12-223.74) while the overall prevalence of smear-positive PTB was 169 per 100, 000 (95% CI: 161.92-175.22) in persons aged 15 years and above (Table 3). Among those screened for PTB in the different districts, the proportions of positive MTB cases varied significantly within the Region, the highest proportion was observed in the Adigrat city (519) as compared to the nil value in four districts (Table 2). As our population-based prevalence survey assessed both existing and new cases of PTB, we identified 16 new and 14 existing cases of PTB. Thus, the ratio of newly detected cases of PTB (active case finding) to those detected through passive case finding and captured by the survey (diagnosed at the health facilities through self-presentation) was about 1.14:1 (16/14) indicating over one undiagnosed TB case in the community for every one smear-positive TB case receiving treatment during the survey period.

**Table 3:** Crude and weighted prevalence of PTB by residence type in Tigray region, Northern Ethiopia, 2011

**Description**	**Crude prevalence of PTB**			**Weighted prevalence of PTB**		
		**95% CI**			**95% CI**	
	**P**	**LCI**	**UCI**	**P**	**LCI**	**UCI**
**Bacteriologically positive PTB**						
Urban	376.65	362.68	390.6	193.49	183.39	203.59
Rural	172.16	163.65	180.7	222.15	212.77	231.53
Total	246.42	238.76	254.1	216.42	209.12	223.74
**Smear-positive PTB**						
Urban	302.77	289.53	316	159.35	148.8	169.89
Rural	132.43	124.78	140.1	170.88	162.39	179.37
Total	197.13	190.06	204.2	168.57	161.92	175.22

*P*, Prevalence; *CI*, Confidence interval; *LCI*, Lower confidence interval; *UCI*, Upper confidence interval.

Among the study subjects, from a total of 5,401 males there were 19 positive PTB cases while from 6,774 females there were 11 positive cases. On the other hand, there were 13 cases from 7,551 rural residents and 17 cases from the 4,624 urban residents ≥ 15 years of age. The prevalence of bacillary TB was higher amongst males (352/100 000; 95% CI: 339.05, 364.52) than females (162/100 000; 95% CI: 153.60, 171.17). The bacteriologically confirmed weighted prevalence of PTB was higher among rural when compared to the urban residents who had an adjusted prevalence of 193 (95% CI: 183.39-203.59) (Table 3).

## Discussion

Prevalence of PTB is an important epidemiological index to measure the burden of the disease in a community. There is variation in the estimated bacteriologically positive and/or sputum smear-positive PTB prevalence figures between this study and other studies within and outside Ethiopia. The estimated overall bacteriologically confirmed prevalence of PTB in the current study was 216 per 100,000 in individuals aged 15 years or above and it was slightly lower than the national estimate of 277 per 100,000 [15]. However, it was within the range of the WHO provisional estimate for 2007 of 146–260 per 100,000 for the region [18] but much higher than the 76 per 100,000 reported in Southwest Ethiopia [7].

The 169 per 100,000 prevalence of sputum smear-positive TB found in our study in persons aged 15 years and above was higher than the national estimate of 108 per 100,000 [15], the 30 per 100,000 in Southwest Ethiopia [7], 78 per 100,000 reported in southern Ethiopia [5] and the 80 per 100,000 documented in northwest Ethiopia [10]. Our finding is more comparable to the reports of other prevalence surveys including the 174 per 100,000 in Northwest Ethiopia [8] and 189 per 100,000 in Addis Ababa, Ethiopia [9].

Reports from African countries indicate that TB prevalence is highly variable in different geographic or regional settings. An overall prevalence of tuberculosis of 870 per 100,000 was reported in adults in Zambia [22]. A study from Eritrea identified a total smear-positive prevalence of 90 per 100,000 in the adult population by using fluorescence microscopy [11]. Another study in Guinea Bissau reported a total TB prevalence of 134 per 100,000 [12]. A TB survey from South Africa had documented a total prevalence of 2,517 per 100,000 [23]. In India, one study reported a higher prevalence of bacteriologically positive TB of 1090 per 100,000 populations in persons aged 15 years and above [14] while another study documented 387 per 100,000 in adult populations [3].

The higher prevalence of PTB reported in the national TB prevalence survey of Ethiopian could probably be the result of using both sensitive chest-x ray screening and symptom screening/inquiry of all study subjects unlike our study where only the head of each household was interviewed for TB symptoms in the family. A study showed that the prevalence of TB may be underestimated by 37% if only symptoms, without CXR, are used to identify TB suspects [24]. Another reason may be due to the fact that the national survey was conducted in the whole country where many regions with higher TB burden than Tigray region might have increased the national prevalence figure. In line with this, a previous report indicated that Tigray region was categorized as a moderate TB burden area compared with other regions in Ethiopia [18]. Moreover, the loss of viability of *M. tuberculosis* bacteria cannot be fully excluded because of the repeated freeze-thaw procedure during sample transport to AHRI. Furthermore, reliable comparison of various prevalence survey results is difficult because of differences in sampling, screening strategies and also the laboratory methods used, both in the smear and culture procedures [25]. Data collection methods, study population, the timing of the study, and related environmental and socioeconomic factors were also considered as important factors affecting the wide country variations in the prevalence of TB [5,26]. The age groups studied may also affect the comparison of the results of a prevalence survey [8].

From a public health perspective, this study clearly indicated that the current TB detection procedure is insufficient to capture all PTB cases occurring in the Region. Hence, another innovative surveillance system needs to be introduced to fully capture and control the transmission of the disease.

This survey found a discrepancy in the urban to rural distribution of PTB. The prevalence of bacteriologically confirmed PTB was higher among rural residents. In agreement with our study, higher prevalence of TB was reported in a rural community in Viet Nam [27] and India [28]. But no statistically significant difference was observed in the national TB prevalence survey of Ethiopia [15]. The higher prevalence of TB in rural areas was attributed to poor performance of anti-tuberculosis services in rural areas due to various reasons such as a lack of awareness of the disease and of available services, difficult terrain resulting in irregular drug supply and poor supervision by programme officials [28]. In Contrary to these reports, higher prevalence of TB was reported in urban compared with rural communities in Zambia [22]. The higher prevalence of PTB in a rural community in our study contradicts previous reports in Ethiopia [5,15] and needs further evaluation.

This study found a higher prevalence of PTB in men in comparison to women. This is consistent with the national prevalence survey in which men had a higher prevalence of 287 per 100,000 population as compared to the 232 in women [15]. Our finding was also in agreement with a report from Guinea Bissau where males had 2.6 higher risk of acquiring TB than females [29]. Another study in India [28] and in Viet Nam [27] also found a similar finding. However, there was no significant difference among men and women in a study conducted in Zambia [22]. In most countries, TB notification rates were found to be higher for men than women even in countries with equal access to health care service [30]. A study related this difference to under diagnosis or poor reporting of TB in women [31]. Another report attributed this difference to either behavioral, socioeconomic, true biological effects, or a combination of all [32]. The higher prevalence of TB in males in our population based prevalence study is the result of a true difference in disease occurrence rather than a difference in access to TB diagnosis and treatment.

## Conclusions

This study found a relatively higher prevalence smear-positive PTB in the region than in the same period nationwide TB survey and identified a significant number of undetected PTB cases. Thus, TB case detection procedure needs to be improved through innovative case finding techniques and intensify TB awareness creation in the population.

## Abbreviations

TB: Tuberculosis; HBC: High burden countries; PTB: Pulmonary tuberculosis; AHRI: Armauer Hansen Research Institute; AFB: Acid fast bacilli; ZN: Ziehl Neelsen; MTB: M. tuberculosis.

## Competing interests

The authors declare that they have no competing interests.

## Authors’ contributions

The authors’ contribution was as described below. GB FE and AA conceived and designed the study. GB TT AG RB and TH performed the study. GB FE WM EH and AA analyzed the data: GB FE and AA wrote the manuscript. All authors read and approved the final manuscript.

## Pre-publication history

The pre-publication history for this paper can be accessed here:

http://www.biomedcentral.com/1471-2334/13/448/prepub
